# Enabling Timely Medical Intervention by Exploring Health-Related Multivariate Time Series with a Hybrid Attentive Model

**DOI:** 10.3390/s22166104

**Published:** 2022-08-15

**Authors:** Jia Xie, Zhu Wang, Zhiwen Yu, Bin Guo

**Affiliations:** School of Computer Science, Northwestern Polytechnical University, 1 Dongxiang Road, Chang’an District, Xi’an 710129, China

**Keywords:** medical intervention, multivariate time series, hybrid attentive model, attention mechanism

## Abstract

Modern healthcare practice, especially in intensive care units, produces a vast amount of multivariate time series of health-related data, e.g., multi-lead electrocardiogram (ECG), pulse waveform, blood pressure waveform and so on. As a result, timely and accurate prediction of medical intervention (e.g., intravenous injection) becomes possible, by exploring such semantic-rich time series. Existing works mainly focused on onset prediction at the granularity of hours that was not suitable for medication intervention in emergency medicine. This research proposes a Multi-Variable Hybrid Attentive Model (MVHA) to predict the impending need of medical intervention, by jointly mining multiple time series. Specifically, a two-level attention mechanism is designed to capture the pattern of fluctuations and trends of different time series. This work applied MVHA to the prediction of the impending intravenous injection need of critical patients at the intensive care units. Experiments on the MIMIC Waveform Database demonstrated that the proposed model achieves a prediction accuracy of 0.8475 and an ROC-AUC of 0.8318, which significantly outperforms baseline models.

## 1. Introduction

Intensive care units (ICU) play a pivotal role in caring for the most severely hospitalized patients [1], where clinicians must anticipate patient care needs according to a set of fast-paced physiological signals, and then provide aggressive life-saving treatments or interventions [2]. To assist clinicians with supporting evidence for timely and accurate medical interventions, an effective approach is analyzing time series which contain representative information related to the health status, e.g., the physiology, the respiratory and the neurological function [3,4,5,6,7,8]. In other words, early event prediction plays an important role in ICUs, and it ensures that hospital staff are prepared for interventions [9].

To provide high level supportive analytics, numerous predictive models and computer-aided diagnostic solutions were proposed [10]. For example, different medical scoring systems (e.g., SOFA, SAPS, APACHE [11]) have been developed to provide computer assisted decision support. Usually, these scoring systems are based on some type of routine physiological measurements followed by logic-based regression techniques. However, these scoring systems are not able to discover the rich semantics of the vital physiological time series and are not well calibrated in predicting results [12].

Although medical scoring systems are still widely used for evaluating various clinical probabilities in the ICUs [13,14,15], machine learning approaches have been attracting more and more attention lately in the literature. In addition to predictive models based on logistic regression, more sophisticated approaches (e.g., random forests and clustering techniques) are employed to improve the predictive performance for early detection of emergency clinical events [15,16,17]. Nevertheless, one main drawback of existing approaches is that they depend on a set of priori features, which are designed manually based on domain knowledge, by considering the multivariate time series as uncorrelated inputs. Thus, they fail to leveraging the complex correlations among multivariate time series for the extraction of latent features [18]. Furthermore, none of these approaches could provide the ability to deal with time-varying data in the ICUs [10].

Recently, deep models [19,20] show powerful data representation and feature extraction advantages, which have been successfully applied to different medical scenarios [21,22,23] and achieved significant performance improvements over traditional models [24,25,26]. For example, convolutional neural network (CNN) is capable of obtaining a compact latent representation [27], while a long short-term memory network (LSTM) can effectively learn long dependencies of time series [28]. Meanwhile, attention mechanisms have shown great promise of providing interpretable learning results [29], while preserving the versatility and flexibility of deep models. Specifically, such deep models have been successfully used for the prediction of ICU interventions, e.g., ventilation, vasopressors, colloid/crystalloid boluses [30,31].

This work aims to predict the impending need of medical interventions (intravenous injection to be specific) by exploring the patient‘s physiological recordings in ICUs. Virtually intravenous administration has become one of the most common interventions in ICUs and emergency settings. Each day, in acute and critical care conditions, over 30% of patients had received intravenous therapy [32], and a wealth of information of each hospitalized patient is recorded through pervasive sensing, including measurement of high-resolution physiological signals (such as respiration rate, pulse, blood pressure, and temperature), complete clinical information in electronic health records, and various laboratory tests. For these acutely ill patients, medical staff are required to make lifesaving decisions under strict time constraints by dealing with a high level of uncertainty in clinical data and a high-volume of complex physiologic signals.

Thus, for this purpose, there are two important issues to be addressed. First, changes in one or more vital signs prior to a serious adverse event are well documented, and early checking of vital signs is key to timely intervention. However, a vast amount of data with disparate types is continuously captured in real-time as patients stay at ICUs, including static variables (such as gender and age), time-varying vital signals (such as electrocardiogram and oxygen saturation), and clinical notes. Therefore, to achieve timely and accurate intervention prediction, this research needs to select a compact but useful collection of vital time series. Second, the characteristics of biomedical signals before serious adverse events can vary drastically, thus it is difficult to build classifiers based on feature engineering. Moreover, to support clinical decision making, an interpretable model is needed, which should provide easy-to-understand predictions. Therefore, considering the complex correlations among multivariate time series, how to build an interpretable prediction model is the second challenge.

**Figure 1:**
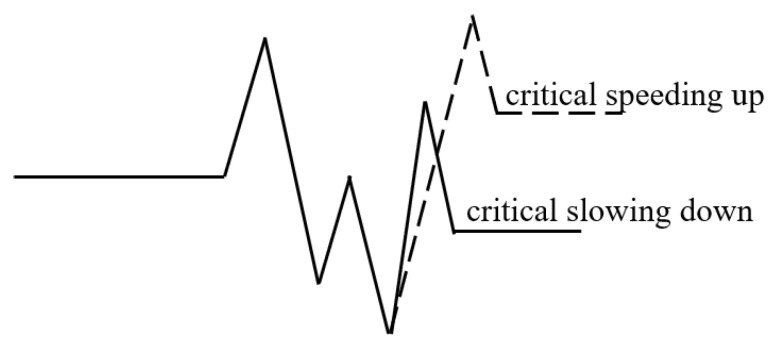
Conceptual illustration of abnormal fluctuations.

To address these challenges, this research designs a Multi-Variable Hybrid Attentive Model (MVHA) to facilitate timely prediction of medical interventions, using fluctuations and trend characteristics of the time series of physiological signals. In other words, the model depends on the assumption that one or more physiological signals should have been altered prior to a medical intervention [33] and reflect the potential life-threatening conditions [34]. In the ICUs, the acute fluctuation and abrupt trend are typical abnormal patterns of the physiological signals, which are driven by the patients’ internal perturbations (e.g., disease). Discovering and understanding such abnormal and hidden implications are critical for timely decision-making in an emergency. On one hand, the time series of vital signs can exhibit oscillations on the order of seconds to min, and significant prognostic values can be obtained by tracking patient specific fluctuations. On the other hand, extra benefits can be acquired by considering the temporal trends of vital signs, which can help improve the prediction accuracy and decrease the false alarm rate. Figure 1 shows a typical abnormal period of a certain signal, which illustrates two kinds of abnormal fluctuations, i.e., abnormal speeding up and abnormal slowing down.

Among the physiological signals recorded in ICUs, ECG is one of the most important vital signs [35]. By analyzing the ECG time series, researchers can not only reveal the respiratory rate, heart rate and variability, but also reduce the false alarm in ICUs [36,37,38]. Thus, ECG provides a good chance for understanding the patient’s physiological status. Recently, a number of models have been developed for end-to-end ECG diagnosis and illustrated a superior performance [39,40,41,42]. However, these models were directly fed with raw ECG waveforms, without exploring the fine-grained temporal fluctuations or trends, which are key to ECG-based medical diagnoses [31], especially for the treatment of acute heart attacks, acute coronary syndromes, and other life-threatening symptoms in ICUs [43,44,45]. Moreover, it should be noted that other physiological signals can be important supplements for ECG-based analysis. As a result, to explore the temporal nature of multiple physiological signals, this study proposes to build a hybrid model by combining the convolutional neural network and the recurrent neural network, aiming to take full advantages of CNN’s ability of extracting local features and LSTM’s capability of mining long dependencies of the time series. Specifically, in this work we mainly consider the following signals, including arterial blood pressure (ABP), peripheral arterial oxygen saturation (SpO_2_), heart rate (HR), pulse, and respiration rate (RESP). Then, to further improve the model’s interpretability, this model incorporates a fluctuation attention mechanism for CNN and a multi-channel trend attention mechanism for LSTM. Based on attentive modeling of the hidden characteristics of multi-variate signals, the work can identify the inputs that have more significant influences on the model’s output.

To sum up, the contributions of this paper are three-fold:

First, to characterize the abnormal pattern of physiological variables more accurately, this work propose a novel hybrid neural architecture by combining a CNN and a LSTM. Particularly, CNN aims to find compact latent features in each wave components, and LSTM is utilized to learn long dependencies of time series to model the overall variation patterns.

Second, to enhance the interpretability of the proposed model, this work designs two attention mechanisms, including a fluctuation attention mechanism for CNN and a multi-channel trend attention mechanism for LSTM. Moreover, this work performs attention fusion across fluctuations and trends of different time series to characterize variation patterns according to their importance.

Third, this study achieve state-of-the-art prediction results in the forward-facing prediction of emergency rescue medications in ICU, which can help ensure hospital staff are prepared for interventions as early as possible.

The remainder of this paper is organized as follows. Section 2 reviews the related work. Section 3 describes the proposed approach in details. Experimental results are presented in Section 5. Finally, Section 4 concludes the paper.

## 2. Related Work

This section will briefly review the related work, which can be grouped into three categories.

### 2.1. ICU Scoring Models

The medical scoring model gives an assessment of the patient’s health status in the form of a score [46], which refer to the clinical severity of the patient. The outcome of forecasting scores can help caregivers be aware of patients at risk and take appropriate actions in advance to prevent these patients from deteriorating [47]. For instance, the sequential organ failure assessment score (SOFA score), which is based on six different scores, is useful in predicting the clinical outcomes of critically ill patients [48]. In the logistic organ dysfunction system (LODS), logistic regression techniques are used to determine severity levels and provided an objective tool for identifying the organ dysfunction level (from 1 to 3) for six different organ systems [14].

Specifically, there are two widely used ICU scoring models at present. The Simplified Acute Physiology Score (SAPS) model calculates the severity of disease for patients admitted to intensive care units, by using 12 routine physiological measurements of the past 24 h [49]. The Acute Physiology And Chronic Health Evaluation (APACHE) model is used to calculate the probability of death independent of diagnosis, based on markers for the extent of the abnormality of 12 common physiological and laboratory values [50].

In general, the outputs of the models are ordinal, i.e., a higher score corresponds to a higher severity. However, all of them are based on fixed time intervals, without considering neither the evolving clinical information nor the non-linear constructed latent features [10,30].

### 2.2. ICU Interventions

Intensive care unit interventions refer to medical treatments given to seriously or critically ill patients who are at risk of conditions that may be potential or established organ failures [51]. Existing studies mainly relate to the content of emergency airway care, respiratory failure and so on [52].

Mechanical ventilation (i.e., assisted respiration) is one of the most common intervention implemented in the intensive care medicine [53]. For instance, a number of studies have been conducted to determine the factors that could help predict the possibility of mechanical ventilation and weaning [54,55,56]. Vasopressor is another commonly used intervention in a medical intensive care unit [57]. For example, Wu et al. [58] used a switching-state autoregressive model to predict the need for a vasopressor. Similarly, to make the intervention model more applicable, unsupervised switching state autoregressive models [9] have been developed by combining waveform recordings with demographic information, aiming to simultaneously provide an in-hospital early detection for five different clinical intervention.

Nevertheless, existing works mainly focus on improving the prediction performance for actionable interventions several hours ahead of onset, and none of them have explored the prediction problem of immediate intravenous injections, which is a core focus of our work.

### 2.3. Deep Learning on ICU Data

Intensive care treatment is highly challenging due to the chitinous generation of a large amounts of heterogeneous health-related data. Thereby, more and more attention is being paid to deep learning based data processing and assistant decision-making, aiming to improve the accuracy of clinical identification and prediction [24,29]. For example, Rajpurkar et al. [59] developed a multi-layer CNN model to detect arrhythmias based on ECG time-series. Similarly, a deep learning based model was built to classify 12 rhythm classes [60], which achieved a state-of-the-art performance.

However, these studies mainly explored the time series of a single vital sign, and could not provide a more comprehensive characterization of the patient’s status in clinical environments (especially in ICUs). A better choice is to fuse multiple simultaneously collected time series with deep models. Recently, a set of models had been proposed to combine vital physiological time series with demographic information (including age, gender, lab test results and so on) to provide clinical predictions [30,61]. Similarly, Lipton et al. [62] had shown promising results using multivariate time series of clinical measurements for learning and prediction.

Nevertheless, the timeliness and interpretability of existing models are still not good enough for the prediction of impending medication intervention needs in ICUs. Therefore, a more effective model is needed, which should be able to provide timely and interpretable predictions, by exploring the fine-grained temporal trends and fluctuations of multivariate time series.

## 3. Methodology

This section describe the proposed multi-variable hybrid CNN-LSTM model, which is mainly composed of a multivariate input processing layer, a hybrid attentive model layer and a predictive output layer.

### 3.1. Overview of MVHA

This subsection first briefly describes the framework of MVHA and introduces the notations used in this article. We denote multivariate physiological signals as S=[G,L], where *G* represents the high-frequency waveforms (such as ECG) and *L* represents the numerical waveforms (such as HR). Aligned with the *i*-th intravenous intervention, we denote multi-channel high-frequency waveforms *G* at time step *t* as: $G_{i}\left(t\right)$ = [$g_{i\_t}^{\left(1\right)}$, $g_{i\_t}^{\left(2\right)}$,…, $g_{i\_t}^{\left(CG\right)}$], where $g_{i\_t}^{\left(cg\right)}$∈*R${}^{ng}$*, 0≤t≤T, *cg* = 1, 2,…, *CG* and *CG* = |$G_{i}$|, *ng* denotes the length of $g_{i\_t}^{\left(cg\right)}$. Similarly, the numerical signals *L* at time step *t* is defined as: $L_{i}\left(t\right)$ = [$l_{i\_t}^{\left(1\right)}$, $l_{i\_t}^{\left(2\right)}$,…, $l_{i\_t}^{\left(CL\right)}$], where $l_{i\_t}^{\left(cl\right)}$∈*R${}^{nl}$*, 0≤t≤T, *cl* = 1, 2,…, *CL* and *CL* = |$L_{i}$|, *nl* denotes the length of $l_{i\_t}^{\left(cl\right)}$. Particularly, *T* represents the time steps used for the prediction of a medical intervention, $g_{i}^{cg}$ is the continuously monitored high-frequency waveform by channel *cg*, and $l_{i}^{cl}$ denotes the numerical sign sampled by channel *cl*. The used notations are summarized in Table 1.

**Figure 2:**
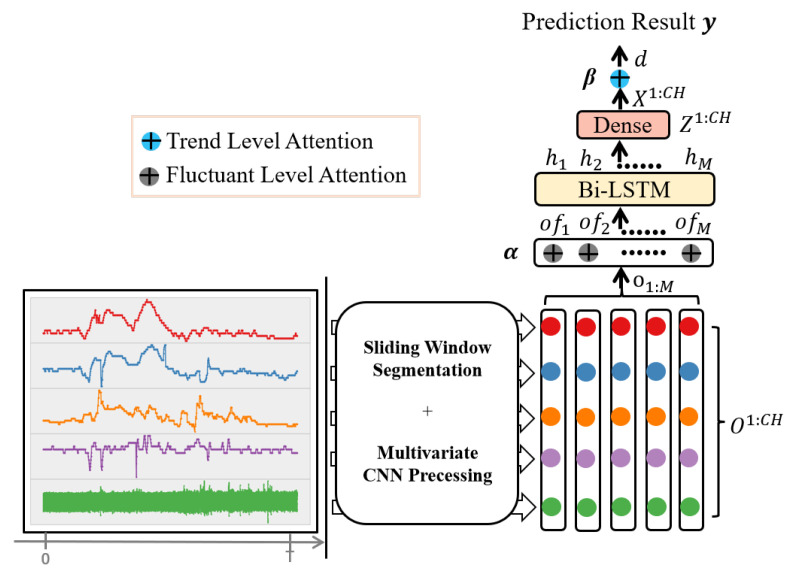
An overview of the MVHA model.

Given a time step *t* and an observation window *W* for the *i*-th intervention, this work takes the observed multivariate time series S(t−W,t] (including both G(t−W,t] and L(t−W,t]) as input, aiming to predict the output value of variable $y_{i}$. With a pre-defined step size, S(t−W,t] is first split into *M* equal length segments: $s_{k}$, 0≤k≤M (e.g., given a step with a length of 1 min, a high-frequency waveform segmentation $g_{k}$ of 125 Hz contains 7500 samples and a numerical waveform $l_{k}$ of 1 Hz contains 60 values). Next, CNN has been applied to these segments to obtain the convolutional output $o_{k}$ and the fluctuant level attention $of_{k}$, followed by a Bi-LSTM that transforms $of_{k}$ into sequentially embedded vectors *H* and *Z*, and then a fully connected layer is adopted to convert *Z* into *X*. After that, this work makes use of the weighted average to integrate *X* = [$x^{\left(1\right)}$,…,$x^{\left(CH\right)}$] (*CH* = |*G*| + |*L*|) across all channels to obtain the trend level attention *d*, which will be concatenated with $tr_{\left(kt\right)}$ (1≤kt≤M−1) and used for prediction. Among them, $tr_{\left(kt\right)}=\left|\rho \left(s_{k+1}\right)-\rho \left(s_{k}\right)\right|$ represents the difference between $s_{k}$ and $s_{k+1}$, where $\rho (s_{k})$ calculates the statistics of segment $s_{k}$ (i.e., max, mean or min). Specifically, to improve the model’s accuracy and interpretability, this study design a two-level attention mechanism (i.e., a fluctuant level attention and a trend level attention, denoted as α and β. Figure 2 depicts the framework of the proposed model.

### 3.2. Details of MVHA

#### 3.2.1. Multi-Variate Attentive Model

To enable effective prediction of medical interventions, this work mainly consider the abnormal wave fluctuations and trends of multivariate signals. To locate such abnormal patterns from signals, this research proposes a hybrid attentive CNN-LSTM model to simultaneously exploit local fluctuations and global trends of physiological waveforms. Specifically, we design two attention mechanisms (i.e., fluctuant level attention and trend level attention) and embed them into the hybrid model. More details of the proposed model are shown in Algorithm 1.
**Algorithm 1** Multi-Variable Hybrid Attentive Model**Input:**     Multivariable physiological signals**Output:**     The predicted result of intravenous intervention: 0 or 11:  s = getSeg(S); // split S into M equal length segments2:  $tr_{\left(kt\right)}$ = getDiff(s); // calculate the difference between $s_{k}$ of all channels3:  P = conv(s); // convert s into features4:  O = sum(P); // output of the CNN layer5:  α = getFluAtt(O); // calculate the fluctuant level attention weights6:  H = biLSTM(αO); // convert αO into recurrent features7:  Z = sum(H); // output of the LSTM layer8:  X = getFull(Z); // convert Z into X through the full connected layer9:  β = getTreAtt(X); // calculate the trend level attention weights10:${\hat{y}}_{i}$ = getPre(βX, $tr_{\left(kt\right)}$); // obtain the prediction result

For a multivariate time series, to exploit the local dependency patterns among different channels, this study adopted convolutional neural networks to encode the time series and map them to the latent space. Formally, the study first split *G* and *L* of *S*(*t* − *W*, *t*] into a sequence of equal length segments. In particular, the segments of *S*(*t* − *W*, *t*] is defined as follows.

(1) $$S\left(t-W,t\right]=\left[\begin{array}{ccc} g_{1}^{1} & \cdots & g_{M}^{1}\\ \vdots & \ddots & \vdots \\ g_{1}^{\left|CG\right|} & \cdots & g_{M}^{\left|CG\right|}\\ l_{1}^{1} & \cdots & l_{M}^{1}\\ \vdots & \ddots & \vdots \\ l_{1}^{\left|CL\right|} & \cdots & g_{M}^{\left|CL\right|}. \end{array}\right]$$

Next, 1-D convolution is applied to the obtained segments to extract features *P = conv(s)*, where *s* stands for $g_{k}^{\left(cg\right)}$ or $l_{k}^{\left(cl\right)}$, *P*∈$R^{U\times J}$, *U* is the number of filters, and *J* is the length of the segment after convolution (a hyperparameter of CNN [29,34]). And then, added $p^{\left(j\right)}$ along the *J* axis together to get the value of *o*, which can be shown as: $o=\sum_{j=1}^{J}p^{\left(j\right)}$, $o\in R^{u}$. The dimension of the *M* segments output was finally fixed at $O\in R^{U\times M}$, in which the first dimension corresponded to the number of filters and the second dimension corresponded to the number of segments. Therefore, the output of the CNN layer is defined as:

(2) $$o=\begin{array}{c} \sum_{j=1}^{J}p^{\left(j\right)} \end{array},$$

where $p^{\left(j\right)}\in R^{U}$, $o\in R^{U}$, and $O\in R^{U\times M}$.

**Fluctuant Level Attentive Layer.** To extract fluctuant level patterns, this study propose a fluctuant-specific weight vector α (with a size of 1 × M) to aggregate the physiological feature maps. Thus, the model obtains better fluctuant level interpretation $of_{k}=\alpha_{k}o_{k}$, where $\alpha_{k}$ represents the weight of the k-th fluctuant level features. Then, to sequentially represent the history information of the physiological time series, we adopt LSTM to characterize the long-term temporal dependencies. Specifically, the LSTM units include a set of gates to control when the information should be maintain in the memory cell, when it should be forgotten and when it should be outputted. For a given time series $X_{t}=\left\{x_{1,t},x_{2,t},\ldots ,x_{k,t}\right\}$ at time *t*, the encoder layer employs the input gate $ig_{t}$, the output gate $og_{t}$ and the forget gate $fg_{t}$ to jointly control the cell state $c_{t}$ and the output $h_{t}$ as follows:

(3) $$ig_{t}=\sigma \left(W_{ix}\cdot x_{t}+W_{ih}\cdot h_{t-1}+b_{i}\right),$$

(4) $$og_{t}=\sigma \left(W_{ox}\cdot x_{t}+W_{oh}\cdot h_{t-1}+b_{o}\right),$$

(5) $$fg_{t}=\sigma \left(W_{fx}\cdot x_{t}+W_{fh}\cdot h_{t-1}+b_{f}\right),$$

(6) $$c_{t}=fg_{t}\cdot c_{t-1}+ig_{t}\cdot tanh\left(W_{cx}\cdot x_{t}+W_{ch}\cdot h_{t-1}+b_{c}\right),$$

(7) $$h_{t}=og_{t}\cdot tanh\left(c_{t}\right),$$

where the group of tensors *W* and *b* are the matrices and bias parameters to be learned during training, $x_{t}$ is the current input, $h_{t-1}$ corresponds to the previous state, and $c_{t}$ is the cell state vector at the current time step. Due to the use of different gates, LSTM can overcome the vanishing gradient problem and capture the long-term dependencies of time series. Specifically, this model use a standard configuration of the bidirectional LSTM network, due to its abilities to capture temporal dependencies. The output of LSTM is denoted as $h_{k}=biLSTM\left(of_{1},of_{2},\ldots ,of_{k}\right)$. Finally, by concatenating the forward and backward outputs, we obtain the sequential encoding features as $H\in R^{J\times M}$.

**Trend Level Attentive Layer.** The trend level attentive layer is designed to obtain a more comprehensive view of the multivariate signals, by fusing attentions across all the channels. First, a fully-connected transformation is performed on the LSTM feature map as follows:

(8) $$X=W_{z}^{T}Z⊙b_{z},$$

where $z=\begin{array}{c} \sum_{k=1}^{M}h_{\left(k\right)} \end{array}$, $Z\in R^{J\times CH}$, $W_{z}\in R^{J\times I}$, $b_{z}\in R^{I}$, and $X\in R^{I\times CH}$. Then, considering that different signal channels play different roles and have various importance, this model introduce a trend-specific weight vector β (with a size of 1×CH) to fuse the trend level attentions as $d=\begin{array}{c} \sum_{k=1}^{CH}\beta^{\left(k\right)}x^{\left(k\right)} \end{array}$. Finally, given the encoded state *d* and the time-varying variable $tr_{kt}^{ch}$, the model can predict a categorical output $y_{i}$ based on multivariate regression as follows:

(9) $${\hat{y}}_{i}=softmax\left(W_{h}^{y_{i}}d+W_{tr}^{y_{i}}tr_{kt}^{ch}+b^{y_{i}}\right).$$

Specifically, the model adopt the cross-entropy loss function as follows:

(10) $$\begin{array}{c} CE=-\sum_{i=1}^{\tilde{N}}y_{i}log{\hat{y}}_{i}, \end{array},$$

where $\hat{N}$ denotes the number of instances in a mini-batch, $y_{i}$ and ${\hat{y}}_{i}$ represent the true label and the predicted label of the i-th instance, respectively.

#### 3.2.2. Hybrid Attention Mechanisms

The above section have described the framework of the proposed model. To further explain the design principle of the model, this subsection will present the details of the proposed hybrid attention mechanisms.

In order to better characterize fluctuation and trend changes, this study imported two attention mechanisms in the proposed model, i.e., a fluctuant attention and a trend attention. To obtain the fluctuant attention vector α and the trend attention vector β, the model design is a two-step neural network. Specifically, the first full connection layer is used to calculate the scores for computing weights, and the second full connection layer is designed to compute the weights with via Softmax activation.

**Figure 3:**
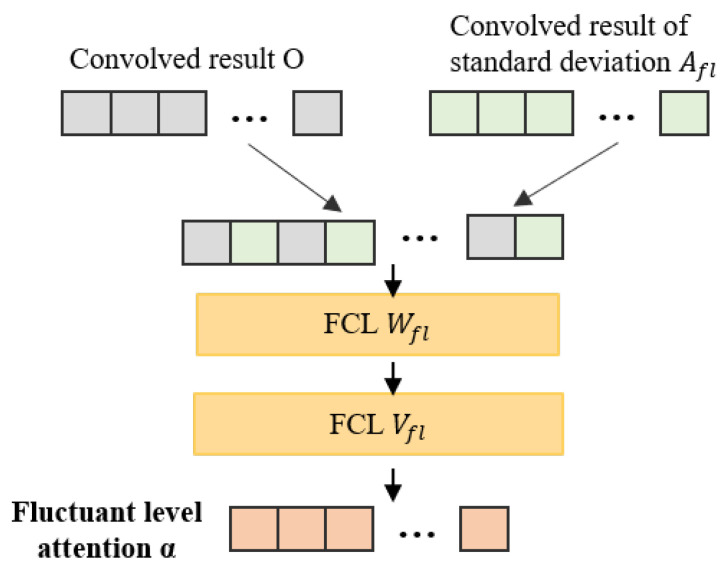
The structure of fluctuant attention mechanism.

**Fluctuant Attention Mechanism.** To characterize fluctuations with attention weights α, the model first compute the standard deviation of each obtained segment *s*, and obtain the fluctuant level knowledge feature vector $A_{fl}=SD\left(S\right)$ as follows:

(11) $$A_{fl}=SD\left(S\right)=\frac{1}{\left|s\right|}\sum {\left(s_{i}-\bar{s}\right)}^{2},$$

where SD(·) calculates the standard deviation of each *s* of the time series *S*. Afterwards, the model concatenates the knowledge features with the output of the CNN layer to obtain the attention weights:

(12) $$\alpha =softmax(V_{fl}^{T}\left(W_{fl}^{T}\left[\begin{array}{c} O\\ A_{fl} \end{array}\right]⊙b_{fl}\right)),$$

where $W_{fl}\in R^{\left(U+E_{fl}\right)\times D_{fl}}$ is the weighted matrix at the first layer, $V_{fl}\in R^{D_{fl}\times 1}$ is the weighted vector at the second layer, $b_{fl}\in R^{D_{fl}}$ is the bias vector, ⊙ denotes an addition with broadcasting, $A_{fl}\in R^{E_{fl}\times M}$, and $\alpha \in R^{M}$. We further present the fluctuant attention in more detail in Algorithm 2. Figure 3 shows the structure of fluctuant attention.
**Algorithm 2** Fluctuant Attention Mechanism.**Input:**     output of the CNN layer *O***Output:**     fluctuant level attention weights1:  $A_{fl}$ = SD(*S*); // calculate the standard deviation of each segment *s* of the time series *S*2:  AttO = getSim(*O*, $A_{fl}$); // calculate the similarity between *O* and $A_{fl}$3:  AttT = getFull(AttO); // convert AttO into AttT through the fully connected layer4:  α = softmax(AttT); // calculate the fluctuant level attention weights

**Trend Attention Mechanism.** Intuitively, signals with significant changes are likely to contain more important information, and should be given more attentions. However, as different channels of the multivariate time series usually have different amplitudes, this study adopts the *min-max scaling* to normalize the time series first, based on which this model further extracts the trend level knowledge feature weights $A_{tr}$ of each channel as:

(13) $$trc^{ch}=max\left|\frac{1}{|s_{k}^{ch}|}\left(\sum s_{k}^{ch}\right)-\frac{1}{|s_{k^{"}}^{ch}|}\left(\sum s_{k^{"}}^{ch}\right)\right|.$$

**Figure 4:**
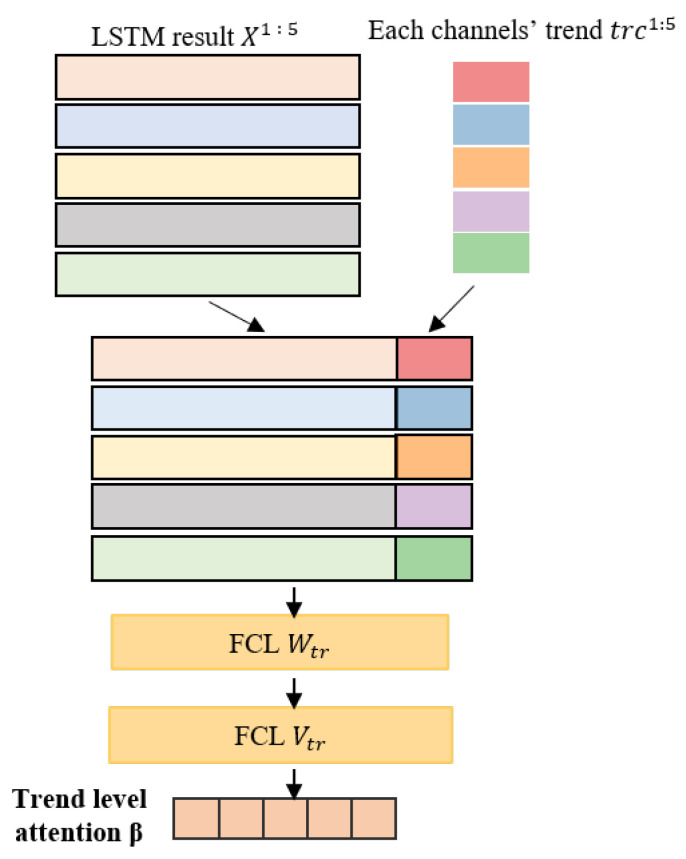
The structure of trend attention mechanism.

Based on the above formula, the model can obtain the trend level knowledge feature vector $A_{tr}=\left[trc^{1},\ldots ,trc^{CH}\right]$, and then calculate the attention weight β as follows:

(14) $$\beta =softmax(V_{tr}^{T}\left(W_{tr}^{T}\left[\begin{array}{c} X^{1:CH}\\ A_{tr} \end{array}\right]⊙b_{tr}\right)),$$

where $W_{tr}\in R^{\left(U\times M+E_{tr}\right)\times D_{tr}}$ and $V_{tr}\in R^{D_{tr}\times 1}$ are the weighted matrix and vector in the first and second layers, respectively. $b_{tr}\in R^{D_{tr}}$ is the bias vector, ⊙ represents an addition with broadcasting, $A_{tr}\in R^{E_{tr}\times CH}$, and $\beta \in R^{CH}$. We further present the proposed trend attention in more detail in Algorithm 3. Figure 4 shows the structure of trend attention.**Algorithm 3** Trend Attention Mechanism.**Input:**     **physiological time series *S*****Output:**     trend level attention weights1:  $ns^{ch}=getNor\left(s^{ch}\right)$; // normalize each channel of the time series2:  $ms_{k}^{ch}=getMean\left(ns_{k}^{ch}\right)$; // calculate the mean of the *k*-th segment $ns_{k}$3:  $ds_{k}^{ch}=getDiff\left(ms_{k}^{ch},ms_{k^{{}^{\prime }}}^{ch}\right)$; // calculate the difference between all the segments4:  $trc^{ch}=getMax\left(ds_{k}^{ch}\right)$; // obtain the maximum value of $ds_{k}^{ch}$5:  AttO = getSim(*X*, $trc^{ch}$); // calculate the similarity between *X* and $trc^{ch}$6:  AttT = getFull(AttO); // convert AttO into AttT through the fully connected layer7:  β = softmax(AttT); // calculate the trend level attention weights

## 4. Experiments

This section first describes the dataset and baseline models used in this work, and then presents the experimental results.

### 4.1. Dataset

To evaluate the performance of the proposed model, this research use the MIMIC-III (Multi-parameter Intelligent Monitoring in Intensive Care) Waveform Database Matched Subset [63]. MIMIC is a publicly available benchmark dataset which contains over 58,000 hospital admissions from approximately 38,600 adults, whose physiological signals were recorded continuously in ICUs. These waveform records include thousands of recordings of waveforms (such as one or more channel of ECG signals) and the time series of vital signs (such as heart and respiration rates). This research chose 18 frequently used rescue intravenous drugs in critical care unit (CCU) [64], which is a special department of the ICU, and got 19,608 experimental records. These medications include sodium nitroprusside, nitroglycerin, dopamine, dobutamine, norepinephrine, milrinone, amiodarone, lidocaine, epinephrine, adenosine, alteplase, esmolol, diltiazem, phenylephrine, hydralazine, nesiritide, procainamide, and isoproterenol.

**Figure 5:**
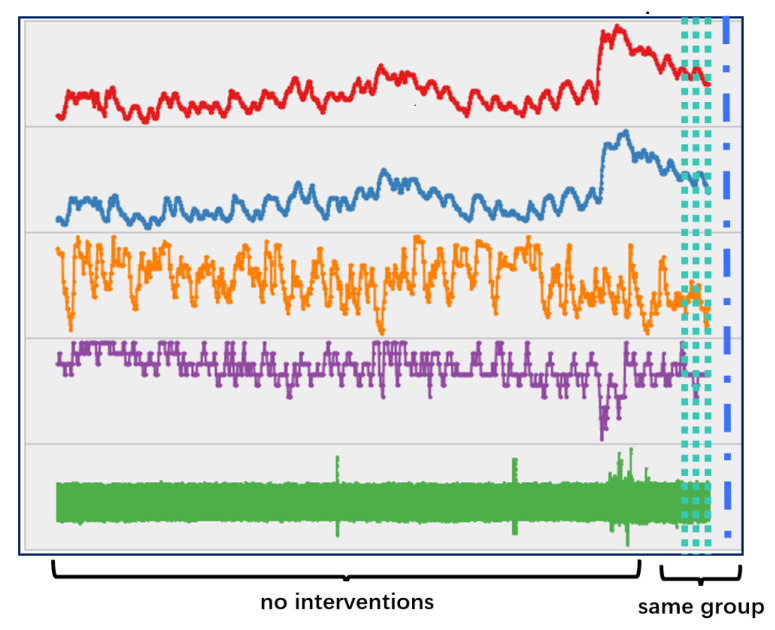
One subject’s multi-channel time series which includes a group of intravenous injections.

In the experiment, this work aimed to predict whether an intravenous injection of the mentioned drugs is needed. Specifically, this research formulates the prediction issue as a binary classification problem, i.e., whether the patient needs an injection within a certain time period. Normally, the medical staff of the emergency treatment in ICUs would inject a variety of drugs into patients in a relatively short time period. Therefore, this work takes all drugs that were injected 2 min before and after a certain time point as the same group. For example, as shown in Figure 5, the subject was given a group of injections, including three doses of norepinephrine and one dose of lorazepam.

Accordingly, this work identified 18,792 groups of intravenous injections. For each injection event, 30 min of time series were extracted from the dataset by taking the event as an endpoint. With the constraint that there should be only one group of intravenous injections in the extracted time series, a total number of 14,465 groups were obtained. The experiment took the first half of each time series as a negative sample and the second half as a positive sample. Specifically, the obtained time series consisted of five vital signs, i.e., heart rate (hr), pulse, respiratory (resp), peripheral capillary oxygen saturation (SpO${}_{2}$) and ECG. Missing values were imputed using piecewise cubic spline interpolation in the experiment.

### 4.2. Experimental Setup and Baseline Models

**Training and Implementation Details.** For the training of CNNs, various numbers of convolutional layers (ranging from 1 to 5) and filters (ranging from 8 to 64) have been tried, with the hyperparameter of stride setting as 1 or 2. Similar to existing studies [29,65,66], this study use batch normalization, rectified linear unit (ReLU) activation and max pooling between convolutional layers to prevent overfitting. Specifically, this model utilize a 3-layer CNN for high-frequency time series (i.e., EEG) with the filter size ranging from 10 to 3, a 2-layer CNN for the other time series with the filter size varying from 5 to 2.

**Figure 6:**
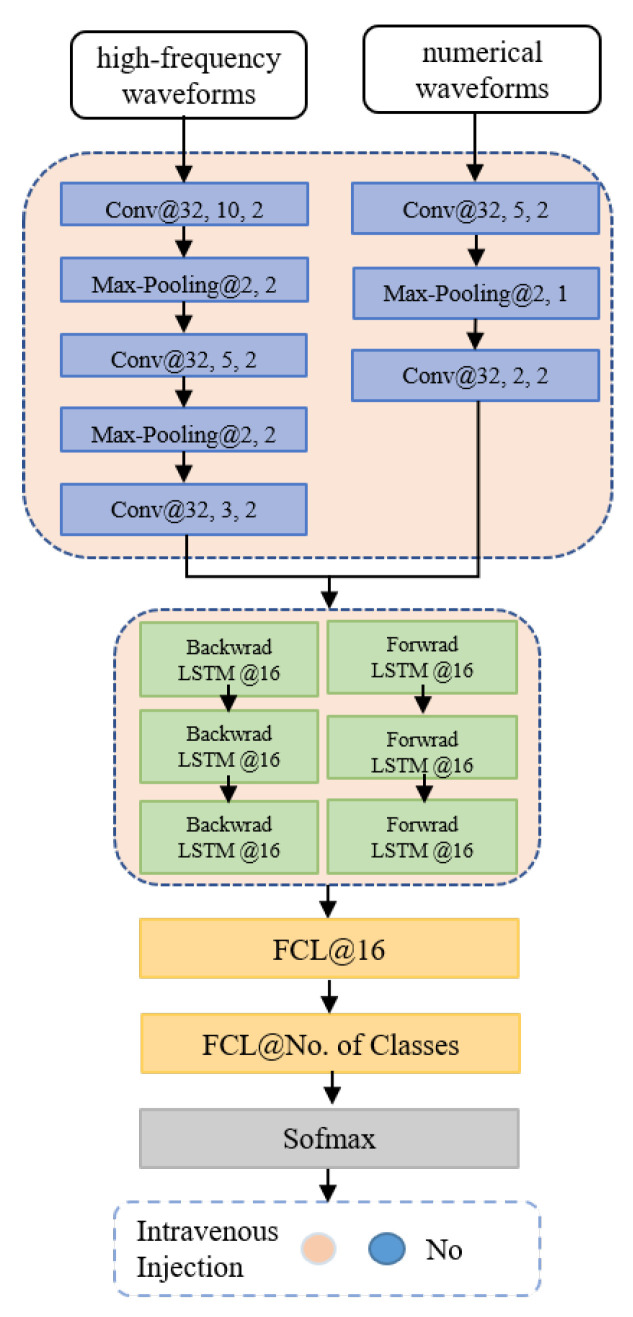
The concrete architecture of the hybrid model. In each layer, the meaning behind symbol ‘@’ indicate the size of the convolution filter, the number of neurons, the stride of the filter, or the size of the pooling layer, the stride of the pooling layer, respectively.

Furthermore, this work explore the Bi-LSTM from one to eight layers and the number of hidden units from 8 to 64. Meanwhile, different configurations are tested, including different mini-batch sizes (16, 32 and 128) and different optimizers (stochastic gradient descent, adagrad and Adam). Specifically, the model used a 3-layer Bi-LSTM by setting the number of hidden units to 16. The model’s initial weights/parameters are given randomly, and the learnable ones are updated in each loop based on the Adam optimizer, with the learning rate of 0.002. The dropout rate is set to 0.5 in the fully connected prediction layer. The model is trained with a mini-batch size of 128 samples, and the dataset is randomly divided into three subsets, i.e., a training set (70%), a validation set(10%) and a test set (20%). In our experiments, all models are implemented with Pytorch 1.1.0 and the used machine is equipped with Intel Xeon E5-2640, 256 GB RAM, 8 Nvidia Titan-X GPU and CUDA 8.0. The workflows of the proposed hybrid CNN-LSTM model is shown in Figure 6.

**Baseline Models.** In this work, different baselines are employed to compare with the proposed model MVHA.

(a) CNN (ECG)—The CNN model is performed on one minute of ECG segments, followed by a fully connect layer and a Softmax layer for prediction;
(b) CNN-LSTM—The vanilla CNN and Bi-LSTM are trained using the full time series, with a fully connect layer and a Softmax layer on the top of the hidden layers;
(c) CNN-FAttn—The CNN model is used to encode all the time series, with the fluctuant level attention mechanism for better representation;
(d) CLSTM-FAttn—The fluctuant level attention mechanism is introduced to the CNN-LSTM model;
(e) CLSTM-TAttn—The trend level attention mechanism is introduced to the CNN-LSTM model.

### 4.3. Experimental Results

The experiment measure the models’ performance based on accuracy (ACC), area under the ROC curve (ROC-AUC) and F1 score.

**Figure 7:**
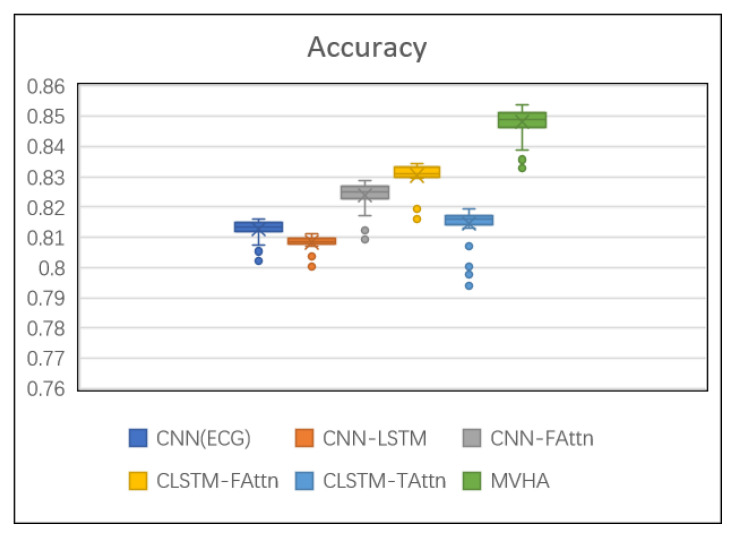
The boxplot diagram of accuracy.

Table 2 reports the performance of each model on the prediction task. The results reflect that the proposed model MVHA outperforms all other models. Meanwhile, all attention-based predictions show better performance than without, which agree with the premise of utilizing the attention mechanisms can distinguish between samples more clearly in result. In order to get a better view of the results, a boxplot graph of the accuracy is shown in Figure 7.

CNN (ECG) has a relatively satisfied classification result and two main reasons are speculated: first, the samples came from CCU which treated patients with severe cardiac diseases, and these acute diseases influence the ECG directly; second, high dense signals contain enough information for completing some certain tasks, and the ability of the designed CNN could utilize these multidimensional inputs efficiently. In other ways, however, its performance was inferior to that of other models (such as CNN-FAttn), perhaps suggesting that ECG needs to be integrated with other time series data for prediction tasks. CNN-FAttn hold all the time series and fluctuant level attention mechanism to improve performance. Particularly, CNN-FAttn surpasses CNN (ECG) by up to 1.5% for ACC, which indicates that the representatives from wider signal sources help in performance improvement.

The rest of the five kinds of models incorporate both CNN and LSTM. CNN-LSTM gives the relatively poorer experiment results compared with other models. It can be explained that a proper short space of waveform from the injection point could provide sufficient contextual information and, if too long, may undermine information already mined from previous search time series. In a further study, the shorter waveforms may be used for such research. Adding multi-channel trend level attention CLSTM-TAttn has higher scores compared to CNN-LSTM, but did not beat CNN-FAttn and CLSTM-FAttn, maybe indicating that whencomparing with the trend variation in a short time, the violent fluctuation of signals seems to be more significant for impending need intravenous injection. Furthermore, it can be found that whatever type of attention models we decided to use, the method can improve the classification performance. Lastly, the proposed model MVHA that incorporates changes from both fluctuation and trends events reaches the best performance on prediction. That is, mining the fluctuation pattern and overall variation trends could retain more useful information for the classification.

**Figure 8:**
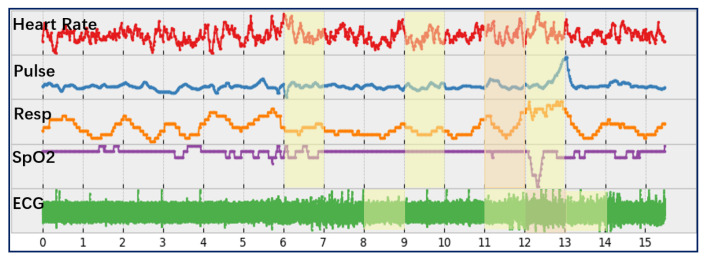
The risk level for an intravenous injection predicted by MVHA. The learned attention cells are highlighted in orange (above 0.15) and yellow (between 0.1 to 0.15).

To validate the interpretability of the proposed attentive model, Figure 8 presents the predicted risk level for an intravenous injection of an unseen patient. Accordingly, this study can find that the patient is predicted to have a higher risk of intervention than average during the 11th–13th min (highlighted cells as yellow and orange). Apparently, a time slice would receive higher attention if it is closer to the time point of an intravenous injection or it contains significant fluctuations, which proved the effectiveness of the proposed fluctuation level attention mechanism.

**Figure 9:**
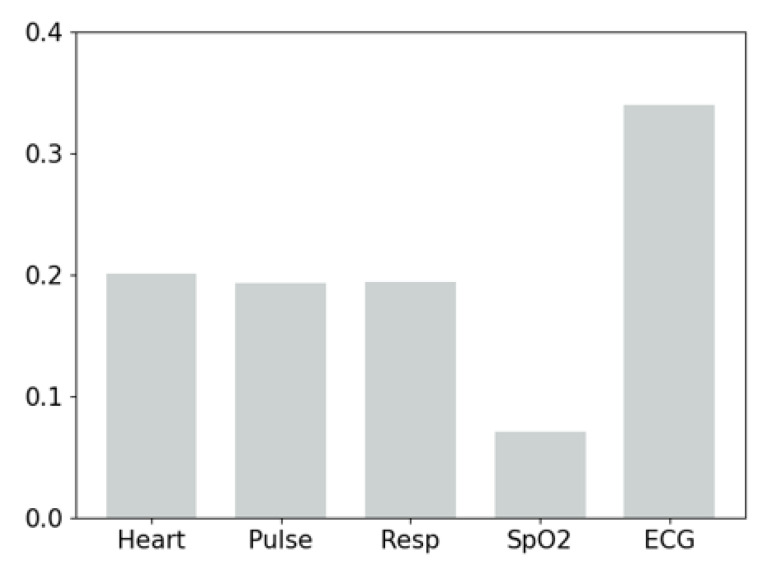
The trend level attention of different channels.

In addition, for the trend level attention (as shown in Figure 9), we find that the ECG channel receives the highest attention weight, the other three channels (i.e., Heat, Pulse and Resp) attract slightly lower attention, and the SpO${}_{2}$ channel has the lowest attention. It indicates that, on the one hand, ECG provides the most important evidence for the prediction of intravenous injections. On the other hand, while high-frequency time series contain abundant information, it is still necessary to take into account other vital signs to enable timely and accurate medical interventions.

## 5. Conclusions and Future Work

This paper proposed a hybrid deep model to enable timely medical intervention by exploring health-related multivariate time series. Specifically, CNNs were utilized to mine local features and LSTM to depict time-dependent features. Furthermore, to improve the interpretability of the prediction result, a two-level attention mechanism (i.e., fluctuant level attention and trend level attention) is developed to focus on key time slices and key channels. MVHA is finally set as 3-layer CNNs for high-frequency time series, 2-layer CNNs for numerical waveforms plus 3-layer Bi-LSTM. Total number of learnable parameters in our model is 3392. Experiments on the MIMIC dataset showed that the proposed model significantly outperformed baseline models. In the future, we plan to extend the proposed model by taking into account multi-modality data, such as medical text and medical image and another possible future direction is to study other kinds of medical interventions. Meanwhile, sparse neural networks, which use what is known as network pruning, would be adopted by a future model in order to reduce the computational load.

Further, in this work, by exploiting multi-channel waveforms, a hybrid attentive neural network was used to predict whether an intravenous injection is needed or not. On the other hand, many correlative references (such as Chen et al. [67]) also demonstrated that a rule-based system in the ICUs could execute decisions much faster with proper training for tagging critical events. However, against the background of this thesis, limitations of rule-based systems are as follows: first, when complex and high-density databases are involved in one decision, it can be hard for humans to try instituting detailed and complete rules; second, if researchers want to make rule-based systems successful, it is important to consider the domain expertise, but that is not fully known at design time. While deep learning is more beneficial for analyzing the data and looking for correlations, rule-based systems are relatively simple and their output is easy for a human to debug. Meanwhile, because using the rule engine‘s data can come in handy in increasing the performance of the deep learning algorithm [68], in future work, neural network and operating rules systems would be considered in tandem, and this could be more beneficial to the framework than replacing rules entirely.

## Figures and Tables

**Table 1:** Notations for MVHA.

Notation	Description
S, s, $s_{k}$	multivariate physiological signals (G and L), one of $g^{\left(cg\right)}$ or $l^{\left(cl\right)}$, the k-th segment in s
G, $g^{\left(cg\right)}$, $g_{k}$	high-frequency waveforms, the cg-th channel in G, the k-th segment in *g*
L, $l^{\left(cl\right)}$, $l_{k}$	numerical waveforms, the cl-th channel in L, the k-th segment in *l*
P∈ $R^{\left(U\times J\right)}$, $p^{\left(j\right)}$ ∈ $R^{U}$	the convolutional features, the j-th column in P
O∈ $R^{\left(U\times M\right)}$, o∈$R^{U}$, $of_{k}$	output of the CNN layer, the sum of $p^{\left(j\right)}$, output of the fluctuant level attention
α, $\alpha_{k}$, β, $\beta^{\left(k\right)}$	weights of the fluctuant level attention, the k-th value in α, weights of the trend level attention, the k-th value in β
H$\in R^{\left(J\times M\right)}$, $h_{k}$	output of the Bi-LSTM layer, the k-th column in H
Z$\in R^{\left(J\times CH\right)}$, z	combination of H, the sum of $h_{k}$
X$\in R^{\left(I\times CH\right)}$, x	output of the fully connected layer, the k-th column of X
$A_{fl}$, $A_{tr}$	feature weights of the fluctuant level attention, feature weights of the trend level attention
d, $tr_{\left(kt\right)}$, ρ($s_{k}$)	output of the trend level attention, difference between $s_{k}$ and $s_{k-1}$, max, mean or min of $s_{k}$
$y_{i}$	prediction result of the i-th segment

**Table 2:** Performance comparison of different models.

	ACC	ROC-AUC	F1
CNN (ECG)	0.8129	0.7917	0.7630
CNN-LSTM	0.8090	0.7845	0.7417
CNN-FAttn	0.8257	0.8119	0.7672
CLSTM-FAttn	0.8314	0.8181	0.7581
CLSTM-TAttn	0.8137	0.7931	0.7617
MVHA	0.8475	0.8318	0.7831

## Data Availability

Not applicable.
